# Validation of the Spanish Version of the Breakthrough Pain Assessment Tool in Patients With Cancer

**DOI:** 10.7759/cureus.69135

**Published:** 2024-09-10

**Authors:** Rocio Gonzalez, Rocio Guillen, Andrés Rocha-Romero, Gabriel Carvajal-Valdy, Leonel Avendaño-Perez, Katherine-R Webber

**Affiliations:** 1 Pain Medicine, Instituto Nacional de Cancerología, Mexico City, Mexico City, MEX; 2 Pain Medicine, Clinica Alive, Mexico City, MEX; 3 Pain Management, Centro Nacional de Control del Dolor y Cuidados Paliativos, San Jose, CRI; 4 Cardiology, Centro Medico ABC, Mexico City, MEX; 5 Palliative Care, Royal Surrey County Hospital, Royal Surrey, GBR

**Keywords:** validation studies, translations, pain assessment, cancer, episodic pain, breakthrough pain

## Abstract

Introduction and background: Assessment of breakthrough pain is essential for adequate management of cancer pain. The Breakthrough Pain Assessment Tool (BAT) has been proven to be a brief, multidimensional, and reliable questionnaire for the assessment of breakthrough cancer pain (BTCP). Currently, there are no validated instruments in Spanish that allow assessing BTCP.

Objectives: The objective of this study was to validate the Breakthrough Pain Assessment Tool - Spanish (BAT-S) version of the BAT in adult patients with cancer.

Methods: The BAT-S was tested in a prospective observational study conducted in adult patients with cancer-related pain and treated in a comprehensive cancer center in Mexico. We conducted a forward-backward translation and cross-cultural equivalence test in the Spanish language. The psychometric properties in patients with cancer were assessed using factor analysis, reliability, and validity. To assess reliability, the Kappa test and the intra-class correlation coefficient were used. For consistency, Cronbach's alpha test was used.

Results. Seventy patients participated in the study; 140 questionnaires were analyzed. The Spanish translation was well accepted by participants. Reliability was comprised between 0.746 for “use of analgesics” and 1.00 for “pain location.” Thirteen of the 14 items had values above 0.8, and 12 above 0.9. Cronbach´s alpha coefficient was 0.7.

Conclusion. This study confirms that BAT-S is a valid and reliable questionnaire to assess breakthrough pain in Mexican patients with cancer. This newly validated tool may be used to facilitate clinical management of primarily Spanish-speaking patients with breakthrough cancer pain.

Key message: This study describes a prospective observational study to assess the validity and reliability of the Breakthrough Pain Assessment Tool in its Spanish version. The results support the use of this newly validated tool to facilitate clinical management of primarily Spanish-speaking patients with breakthrough cancer pain.

## Introduction

Cancer is one of the leading causes of morbidity and mortality worldwide. The incidence is expected to increase in the future [1,2]. Prevalence of pain is high in cancer patients depending on disease stage and specific histology: a meta-analysis reported a 55% pain prevalence rate during anticancer treatment and 66% in advanced, metastatic, or terminal disease [3]. Cancer pain is an unrelenting symptom that negatively alters the quality of life, and the quality of life itself can alter sleep patterns and reduce physical activity and mental health [4-6]. The mainstay of cancer pain treatment involves pharmacological methods, and in selected cases, radiotherapy may have a role; nonetheless, some patients benefit from invasive techniques that have demonstrated effectiveness in specific cases, such as nerve blocks, vertebral augmentation, or intrathecal drug delivery systems [7,8]. Breakthrough pain has been defined as a transient exacerbation of pain, produced spontaneously or associated with a specific triggering factor, predictable or unpredictable, even though the background pattern is stable and well-controlled [9,10].

This has a negative impact on general activities and is related to difficult management [11-13]. Breakthrough cancer-related pain is highly prevalent: a systematic review found a 59.2% overall pooled prevalence with higher rates in patients from palliative care [14,15]. Specific assessment tools for the assessment of breakthrough pain have been validated in English, such as the Alberta Breakthrough Pain Assessment Tool (ABPAT) [16] and the Breakthrough Pain Assessment Tool (BAT) [17]. The BAT scale has been previously translated and validated in Dutch and Korean but has yet not been translated and validated for the Spanish-speaking population [18,19]. This project aimed to translate a clinically useful breakthrough pain assessment tool in cancer patients; we, therefore, have developed a validation study for the BAT scale in Spanish (BAT-S).

## Materials and methods

The process of cross-cultural adaptation from the original reference of the BAT-S scale [17] was conducted according to international guidelines with two forward and backward translations. The process of translating the text into Spanish and validating it on the BAT-S scale was carried out with approval from the original author (Katherine Webber). A direct English-Spanish translation of the BAT scale was performed by two certified translators, and a single version was agreed upon by a consensus of cancer pain experts. A pilot test was carried out in a group of healthy subjects (n = 20); every patient completed the questionnaire under supervision, and the items were assessed by the clinician in order to evaluate if the questions were challenging to answer, difficult to understand, confusing, or shocking. As a result, we reformulated one question, and remarks from this test were included in a new version according to expert consensus. This second version was evaluated in hospitalized patients with cancer (n = 20) who were able to understand and answer it. This new instrument, BAT-S, was afterward validated on a broader population.

Study population

We included patients older than 18 years old and diagnosed with cancer-related pain, according to a pain specialist, followed at the pain clinic of Instituto Nacional de Cancerología (INCan), a comprehensive cancer center in Mexico City. The study period was between November and December 2017. Patients with marked cognitive dysfunction who were unable to answer the questionnaire or unwilling to participate in the study were excluded. Informed consent was required for study participation, and the study protocol was performed after approval of the ethical and research committee by the Institutional Review Board of the National Cancer Institute (017/025/CDI)(CEI/1182/17).

The sample size was calculated by considering five patients for each item on the BAT scale (n = 70). Tests were performed at the pain clinic, and a follow-up retest was performed four days later.

Statistical analyses

Non-numeric elements were expressed by simple frequencies and percentages, whereas numerical items were summarized as medians and interquartile ranges due to their non-normal distribution. The reliability of the BAT-S test was assessed using the Kappa test (non-numerical items) and the intraclass correlation coefficient for numerical items. A value equal to or greater than 0.80 in both tests was considered reliable. The consistency of the BAT-S test was evaluated using Cronbach's alpha test, and a value greater than 0.70 was considered acceptable. All statistical analyses were performed using STATA v.12.1 (Version 22.0. Armonk, NY: IBM Corp).

## Results

Seventy patients with cancer pain (100%) were included, and 140 questionnaires (100%) were completed. Forty-nine (70%) were men, and twenty-one (30%) were women. The median age was 52 years. Regarding educational status, 21 (30%) had elementary education, another 21 (30%) had middle school, 20 (28.6%) had high school, 7 (10%) had a college education, and 1 (1.4%) had postgraduate studies. Primary neoplasms were breast cancer in 18 (25.7%), genitourinary system in 16 patients (22.9%), and digestive tract cancer in 14 (20%) patients. The most common type of pain was visceral pain in 22 (31.4%) patients, followed by mixed pain in 20 (28.6%) patients and bone pain in 11 (15.7%). Results are summarized in Table 1.

**Table 1:** Characteristics of participants IQR: interquartile range.

Characteristics	Total (%) n=70
Sex	
Male	49 (70)
Female	21 (30)
Median age (years) ± IQR	52 ± 20
Educational status	
Elementary school	21 (30)
Middle school	21 (30)
High school	20 (28.6)
Graduate	7 (10)
Postgraduate	1 (1.4)

Regarding breakthrough cancer pain (BTCP) frequency, 37.9% of reported episodes one or two times a day. Seventy-eight percent mentioned presenting aggravating factors of pain, and 92.1% reported pain extenuating factors. In 30% of the subjects, the pain lasted between 5 and 15 minutes. The severity scores of the worst pain, typical pain severity, pain distress, and pain disability averaged 8, 6, 6, and 7, respectively, on a scale of 0 to 10 (Table 2).

**Table 2:** Breakthrough Pain Assessment Tool scale results Number (%) or median ± interquartile range are shown.

Variable	Median (%)
Pain characteristics	
1. Pain location	
In front	82 (58.6)
Behind	40 (28.6)
Both sides	18 (12.9)
2. Pain frequency	
<1 times/day	24 (17.1)
1–2 times/day	53 (37.9)
3–4 times/day	29 (20.7)
>5 times/day	34 (24.3)
3. Pain aggravating factors	110 (78.6)
4. Pain extenuating factors	129 (92.1)
5. Pain duration	
<5 min	27 (19.3)
5–15 min	42 (30)
15–30 min	20 (14.3)
30–60min	15 (10.7)
>60 min	36 (25.7)
6. Worst pain intensity	8 ± 3
7. Usual pain intensity	6 ± 3.5
8. Anguish level due to pain	6 ± 6
9. Pain disability degree	7 ± 5.5
Painkillers	
10. Painkillers use	127 (90.7)
11. Painkillers effectiveness	8 ± 5
12. Time to get the painkiller effect	
No effect	21 (15)
0–10 min	28 (20)
10–20 min	48 (34.3)
20–30min	24 (17.1)
>30 min	19 (13.6)
13. Painkillers side effects	75 (53.6)
14. Discomfort due to side effects	1 ± 8

Ninety percent of the subjects reported using painkillers, with a median effectiveness of 8. The time for painkillers to have an effect was 10-20 min in 34.3% of the cases. A total of 53.6% of the patients reported adverse effects from painkillers (Table 2).

Reliability and consistency

The kappa and intraclass correlation coefficients for reliability were found between 0.746 for the “use of painkillers” item and 1.00 for the “location of pain” item. Thirteen of the 14 items presented values above 0.8 and 12 of the 14 items presented values above 0.9.

**Figure 1:**
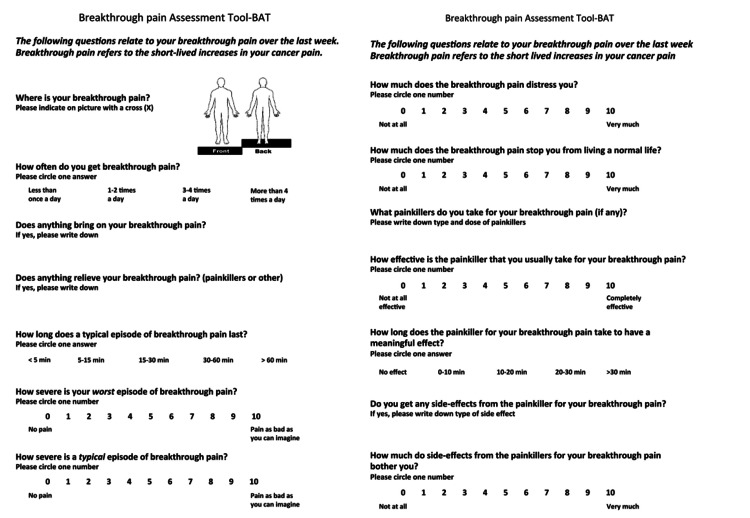
Original version of Breakthrough Pain Assessment Tool (BAT) Development and Validation of the Breakthrough Pain Assessment Tool (BAT) in Cancer Patients, by author K. Webber, 2014, Journal of Pain and Symptom Management. Vol. 48, No. 4. [17] Reprinted with permission.

The consistency values measured with Cronbach's alpha ranged between 0.625 and 0.713. Numerical items are shown in Figure 1. Table 3 summarizes the reliability and consistency results.

**Table 3:** Breakthrough Pain Assessment Tool scale reliability Number (percentage) or median ± interquartile range are shown. Reliability values by kappa test (categorical variables) and intraclass correlation coefficient (numerical variables) with 95% confidence intervals (95% CI) were calculated. Overall Cronbach’s alfa 0.6896 (95% CI 0.6148-0.7644).

Variable	1st application	2nd application	Reliability	Cronbach’s alpha
Number of subjects	70	70	-	-
Pain characteristics				
1. Pain location				
In front	41 (58.6)	41 (58.6)	NA	NA
Behind	20 (28.6)	20 (28.6)	NA	NA
Both sides	9 (12.9)	9 (12.9)	1 (0.821–1)	0.684
2. Pain frequency				
<1 times/day	12 (17.1)	12 (17.1)	NA	NA
1-2 times/day	26 (37.1)	27 (38.6)	NA	NA
3-4 times/day	15 (21.4)	14 (20)	NA	NA
>5 times/day	17 (24.3)	17 (24.3)	0.941 (0.802–1)	0.664
3. Pain aggravating factors	54 (77.1)	56 (80)	0.915 (0.682–1)	0.695
4. Pain extenuating factors	64 (91.4)	65 (92.9)	0.901 (0.668–1)	0.704
5. Pain duration				
<5 min	13 (18.6)	14 (20)	NA	NA
5–15 min	21 (30)	21 (30)	NA	NA
15–30 min	10 (14.3)	10 (14.3)	NA	NA
30–60min	7 (10)	8 (11.4)	NA	NA
>60 min	19 (27.1)	17 (24.3)	0.908 (0.785–1)	0.646
6. Worst pain intensity	8 ± 3	8 ± 3	0.971 (0.954–0.982)	0.646
7. Usual pain intensity	6 ± 3	5 ± 3	0.881 (0.809–0.926)	0.625
8. Anguish level due to pain	6.5 ± 6	6 ± 7	0.96 (0.936–0.975)	0.636
9. Pain disability degree	7 ± 6	6 ± 5	0.964 (0.942–0.978)	0.635
Painkillers				
10. Painkillers use	63 (90)	64 (91.4)	0.746 (0.512–0.979)	0.695
11. Painkillers effectiveness	8 ± 5	8.5 ± 5	0.938 (0.9–0.961)	0.713
12. Time to get the painkiller effect				
No effect	10 (14.3)	11 (15.7)	NA	NA
0–10 min	14 (20)	14 (20)	NA	NA
10–20 min	24 (34.3)	24 (34.3)	NA	NA
20–30 min	12 (17.1)	12 (17.1)	NA	NA
>30 min	10 (14.3)	9 (12.9)	0.908 (0.786–1)	0.700
13. Painkillers side effects	38 (54.3)	37 (52.9)	0.914 (0.68–1)	0.683
14. Discomfort due to side effects	1.5 ± 7	0.5 ± 8	0.965 (0.944–0.978)	0.679

## Discussion

This is a prospective, observational, single-center study that aimed to culturally adapt and validate the BAT (see Appendices, Figure 1) into the Spanish language. BAT-S demonstrated acceptable levels of reliability: test-retest reliability (>0.8), as well as internal consistency, was similar to three earlier studies [17-19]. Therefore, the BAT-S seems to be a reliable, practical, and valid questionnaire for breakthrough pain assessment in patients with cancer.

We demonstrated a high consistency with a 0.7 Cronbach’s alpha, similar to previous studies [17-21]. Items with the highest consistency were “pain relief," “analgesic effectiveness," and “time for analgesic effect." Those of lesser consistency were “severity of typical pain,” “distress,” and “disability.” It draws attention that these same three items had an opposite result in the validation of the Korean scale, resulting in the highest consistency.

**Figure 2:**
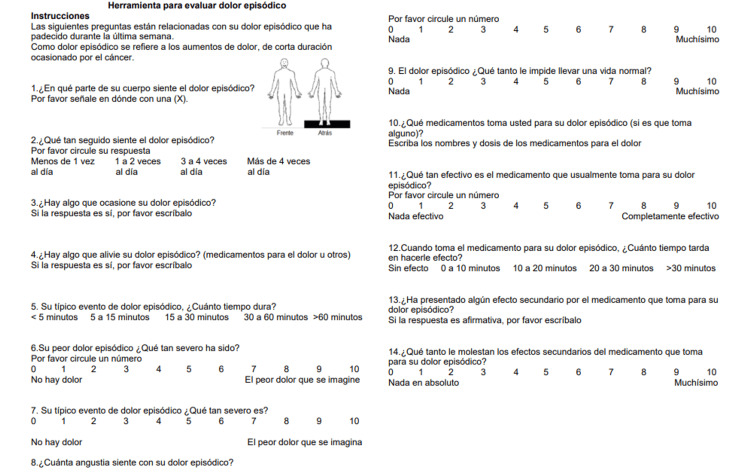
Breakthrough Pain Assessment Tool in Spanish (BAT-S)

We consider that the variation in consistency of the items measured between the validated scales of the three languages may have been influenced by three main factors. First, the retest was applied at different times; in our study, it was carried out after four days; in the original in English and the Dutch validation, it was done 24 hours after the first test and in the Korean language from 24 hours to 7 days. Second, this test was performed in an outpatient setting (where the analgesic treatment is usually modified), compared to the validation in English, Korean and Dutch, for which the participants were either only hospitalized or a combination of outpatient and hospitalized patients. Finally, the BAT-S was adapted using simple images to best clarify instructions (Appendices, Figure 2). Nevertheless, there are some differences with the Portuguese study [20]. First, it was performed in eight hospitals. Although the sample size was also small, similar to our study, this limitation does not allow for testing the underlying structure with a confirmatory factor analysis. On the other hand, the French version [21] included a single academic hospital, the same as ours, and the sample had differences from the original validation sample, such as more patients with impaired performance status and mixed cancer pain, leading to differences from the original.

Patient-reported outcomes are now regarded as essential components in making informed treatment decisions [22]. There is a notable shift towards incorporating patient preferences and symptoms into the decision-making process rather than solely focusing on patient comorbidities and the drug toxicity profile [23].

This study nonetheless has some limitations: It was developed in a single high-specialty comprehensive cancer center, and the population of this pain clinic may be biased toward cases that are more complex. Therefore, management may not be representative of that in other pain clinics. The sample size does not allow for testing the underlying structure with a confirmatory factor analysis. Additionally, the design does not allow for investigating responsiveness to treatment changes. These issues should be addressed in future studies.

## Conclusions

In conclusion, this study confirms and extends the psychometric validation of the BAT to a new cultural context. The BAT-S is a valid and reliable questionnaire for assessing breakthrough pain in patients with cancer. This newly validated tool may be used to facilitate the tailoring of pain management for primarily Spanish-speaking communities, promoting its diffusion and use by patients, researchers, and clinicians, allowing for a personalized approach.

We are currently developing a protocol aimed at identifying the prevalence of breakthrough pain in cancer patients. The secondary objectives of this study will include the examination of the most common presentations of breakthrough pain, as well as the treatments prescribed for each pain scenario. The primary focus of this study is to validate the tool that will facilitate the progression to the subsequent phase.
